# Landscape of Biomarkers in Non-small Cell Lung Cancer Using Comprehensive Genomic Profiling and PD-L1 Immunohistochemistry

**DOI:** 10.3389/pore.2021.592997

**Published:** 2021-03-11

**Authors:** Richard S. P. Huang, Eric Severson, James Haberberger, Daniel L. Duncan, Amanda Hemmerich, Claire Edgerly, N. Lynn Ferguson, Garrett Frampton, Clarence Owens, Erik Williams, Julia Elvin, Jo-Anne Vergilio, Jonathan Keith Killian, Douglas Lin, Samantha Morley, Deborah McEwan, Oliver Holmes, Natalie Danziger, Michael B. Cohen, Pratheesh Sathyan, Kimberly McGregor, Prasanth Reddy, Jeffrey Venstrom, Rachel Anhorn, Brian Alexander, Charlotte Brown, Jeffrey S. Ross, Shakti H. Ramkissoon

**Affiliations:** ^1^ Foundation Medicine, Inc., Morrisville, NC, United States; ^2^ Foundation Medicine, Inc., Cambridge, MA, United States; ^3^ Wake Forest Comprehensive Cancer Center, Wake Forest School of Medicine, Winston-Salem, NC, United States; ^4^ Department of Pathology, Wake Forest School of Medicine, Winston-Salem, NC, United States; ^5^ Department of Pathology, State University of New York (SUNY) Upstate Medical University, Syracuse, NY, United States

**Keywords:** biomarkers, non-small cell lung cancer, comprehensive genomic profiling, PD-L1, immunohistochemistry

## Abstract

Comprehensive genomic profiling (CGP) and immunohistochemistry (IHC) are important biomarker tools used for patients with non-small cell lung cancer (NSCLC) given the expanding number of standard-of-care therapies that require companion diagnostic testing. We examined 9450 NSCLC real-world patient samples that underwent both CGP and programmed death-ligand 1 (PD-L1) IHC to understand the biomarker landscape in this patient cohort. By assessing National Comprehensive Cancer Network (NCCN)-recommended biomarkers including genomic alterations, tumor mutational burden (≥10 mutations/Mb cut-off), and PD-L1 expression (Tumor Proportion Score (TPS) ≥ 50% cut-off), we show that CGP + PD-L1 IHC yielded potentially actionable results for 70.5% of the 9,450 patients with NSCLC. Among the remaining 29.5% (2,789/9,450) of patients, 86.7% (2,419/2,789) were potentially eligible for another biomarker-associated therapy and/or clinical trial based on their genomic profile. In addition, in the PD-L1^TPS≥50%^ disease subset, *BRAF* mutations, *MET* mutations, *MET* amplifications, and *KRAS* mutations were significantly enriched; and in the PD-L1^TPS<50%^, *EGFR* mutations, *ERBB2* mutations, *STK11* mutations, and *KEAP1* mutations were enriched. These findings highlight the improved clinical utility of combining CGP with IHC to expand the biomarker-guided therapeutic options available for patients with NSCLC, relative to single biomarker testing alone.

## Introduction

An estimated 228,150 individuals in the United States were diagnosed with lung cancer in 2019, with non-small cell lung cancer (NSCLC) accounting for approximately 85% of these patients [1]. As the availability of targeted therapies for patients with NSCLC has increased, the importance of histologic subtypes of NSCLC has waned, while categorization of these tumors by genomic and protein expression has become impactful in determining the therapy predicted to have the highest clinical benefit.

The increasing prominence of diagnostic assays used to identify predictive biomarkers accompanying specific therapies, known as companion diagnostics (CDx), is evidenced by the acceleration of oncologic drug approvals with a CDx requirement in recent years [2]. The 2019 National Comprehensive Cancer Network (NCCN) guidelines highly recommend testing of patients with NSCLC for *EGFR* and *BRAF* mutations; *ALK*, *ROS1*, and *NTRK* rearrangements; and programmed death-ligand 1 (PD-L1) expression levels; and recommends *RET* rearrangement; *ERBB2* and *MET* mutations; *MET* amplifications [3]. All of these genomic alterations are potentially targetable based on positivity of these alterations. For examples, *EGFR* mutations have been shown to have sensitivity to EGFR tyrosine kinase inhibitors (TKI) such as erlotinib, and gefitinib; *BRAF* mutations have been shown to be sensitive to dabrafenib plus trametinib in metastatic nonsquamous NSCLC; *ALK* and *ROS1* rearrangements have been shown to respond well to numerous tyrosine kinase inhibitors (TKI) such as crizotinib and certinib; *NTRK* fusions have been responsive to larotrectinib and entrectinib; and responsiveness to multiple therapies have also been found in numerous clinical studies of patients with *RET* rearrangement; *ERBB2* and *MET* mutations; and *MET* amplifications [3]. In addition, although *KRAS* is typically not a biomarker used to identify patients eligible for targeted therapy, *KRAS* mutations have been associated with lack of response for *EGFR* TKIs and promising targetable therapies to *KRAS* mutations such as G12C are being investigated [4, 5].

The immunotherapy biomarker landscape is increasingly complex and changing rapidly. In NSCLC, DAKO 22C3 was the first assay approved as a CDx for pembrolizumab in NSCLC at a tumor portion score (TPS) of 50, and the CDx cut-off has since been lowered to a TPS of ≥1 [6]. In addition, in May 2020, DAKO 28-8 was approved as a CDx for opdivo and VENTANA SP142 was approved as a CDx for atezolizumab in NSCLC. Other relevant biomarkers for immunotherapy eligibility include MSI (microsatellite instability) testing and tumor mutational burden testing (TMB), both of which can be measured by comprehensive genomic profiling (CGP). For MSI testing, microsatellite instability-high (MSI-H) status or loss of MMR (mismatch repair) markers by immunohistochemistry allows a patient to be potentially eligible for pembrolizumab based on MSI being a pan-tumor companion diagnostic for pembrolizumab [7]. In June 2020, the FDA approved the FoundationOne®CDx Assay for pembrolizumab at a TMB ≥ 10 mutations/Mb cut-off in solid tumors based on the KEYNOTE-158 clinical trial [8, 9]. Adding to the complexity are potential resistance biomarkers such as *STK11*, *KEAP1*, and *EGFR* for checkpoint inhibitors (CPIs) [10–12].

In this study, we performed a survey of biomarkers used in NSCLC real-world samples assessed by comprehensive genomic profiling (CGP) combined with PD-L1 immunohistochemistry (IHC) to understand the landscape of the genomic and protein expression biomarkers in NSCLC. In addition, we examined the relationship between PD-L1 and TMB to better understand the interplay of these biomarkers in the context of immunotherapy.

## Materials and Methods

### Patient Cohort

This study was approved by the Western Institutional Review Board Protocol No. 20152817. We performed a retrospective analysis of 9450 NSCLC patient samples that were tested with both CGP and PD-L1 IHC. Formalin-fixed, paraffin-embedded (FFPE) tissue of either whole section samples, biopsies, or cytology specimens were received as a paraffin block or unstained slides from outside institutions with accompanying pathology reports and clinical information such as the age and sex of the patients. All specimens received were confirmed to be NSCLC by a board-certified pathologist based on microscopic examination of a hematoxylin and eosin (H&E) stained slide.

### DAKO PD-L1 IHC 22C3 PharmDx Assay

All PD-L1 testing was performed using the DAKO PD-L1 IHC 22C3 pharmDx assay per manufacturer’s instructions in a Clinical Laboratory Improvement Amendments (CLIA)-certified and College of American Pathologists (CAP)-accredited reference laboratory (Foundation Medicine, Morrisville, NC). PD-L1 IHC slides were interpreted by board-certified pathologists using the Tumor Proportion Score (TPS) scoring method where TPS = Number of PD-L1 positive tumor cells/(Total number of PD-L1 positive + PD-L1 negative tumor cells) [13]. Per DAKO’s pathologist interpretation guidance for the TPS score, we scored partial or complete cell membrane staining (≥1+ in intensity) that is perceived distinct from cytoplasmic staining [13]. In this analysis, we used a TPS ≥ 50% cut-off which is a more conservative to account for the rapidly evolving nature of these biomarkers.

### Comprehensive Genomic Profiling Using FoundationOne^®^CDx

CGP was performed using the Food and Drug Administration (FDA) approved FoundationOne®CDx assay (Foundation Medicine, Cambridge, MA) using previously described methods [14]. FoundationOne®CDx uses a hybrid capture methodology and detects base substitutions, insertions/deletions, and copy number alterations in 324 genes and select gene rearrangements in 36 genes, as well as provides results for TMB and MSI [15, 16]. As per the recent FDA approval for TMB for pembrolizumab in solid tumors, we chose to use TMB ≥ 10 mutations/Mb as our cut-off in this study.

### Data Analysis

We examined all recommended genomic biomarkers suggested by the 2019 NCCN guidelines for NSCLC including *EGFR*, *BRAF*, *ERBB2*, and *MET* mutations; *ALK*, *ROS1*, *NTRK*, and *RET* rearrangements; and *MET* amplifications. Also, we examined *KRAS* mutations due to its negative predictive value for *EGFR* TKIs and potential positive predictive value for KRAS inhibitors. Lastly, we examined *STK11*, *KEAP1*, and *EGFR* for their role as potential resistance mutations for CPIs.

We performed Fisher Exact Test to examine prevalence differences of these genomic biomarkers between the PD-L1 TPS ≥ 50 and TPS < 50 disease subsets. *p*-value was adjusted for multiple comparisons using the Bonferroni method and *p* < 0.05 was considered significant with Bonferroni correction [17]. Sex and age of the PD-L1 TPS ≥ 50 and PD-L1 < 50 disease subsets were also compared with Fisher Exact Test and ANOVA, respectively.

In addition, we examined the relationship of PD-L1 expression and TMB. Lastly, we examined the PD-L1 high positive rate between the NSCLC adenocarcinoma subtype when compared to other subtypes by performing a *t*-test.

## Results

### Patient Cohort

A total of 9,450 consecutive NSCLC patients with concurrent CGP and PD-L1 IHC was extracted from our research database. 51.4% (*n* = 4,859) of the patients were female with a mean age of 67.8 years old and a median age of 68.0 years old. No significant difference was found between the age and sex of the PD-L1 TPS ≥ 50 and PD-L1 < 50 cohort (Fisher Exact Test, 0.067; ANOVA, 0.294; respectively) as shown in Table 1.

**TABLE 1:** Demographics of NSCLC PD-L1 TPS ≥ 50 and PD-L1 TPS < 50 cohort.

	Total (*n* = 9,450)	PD-L1 ≥ 50 (*n* = 2,885)	PD-L1 < 50 (*n* = 6,565)	*p*-value
Female	51.4% (4,859)	50.0% (1,442)	47.9% (3,145)	0.067^a^
Male	48.5% (4,587)	50.0% (1,443)	52.0% (3,416)	
Age (mean, year old)	67.8	67.7	67.9	0.294^b^
Age (median, year old)	68.0	68.0	68.0	

^a^ Fisher Exact Test.

^b^ ANOVA.

### Landscape of Biomarkers

**FIGURE 1:**
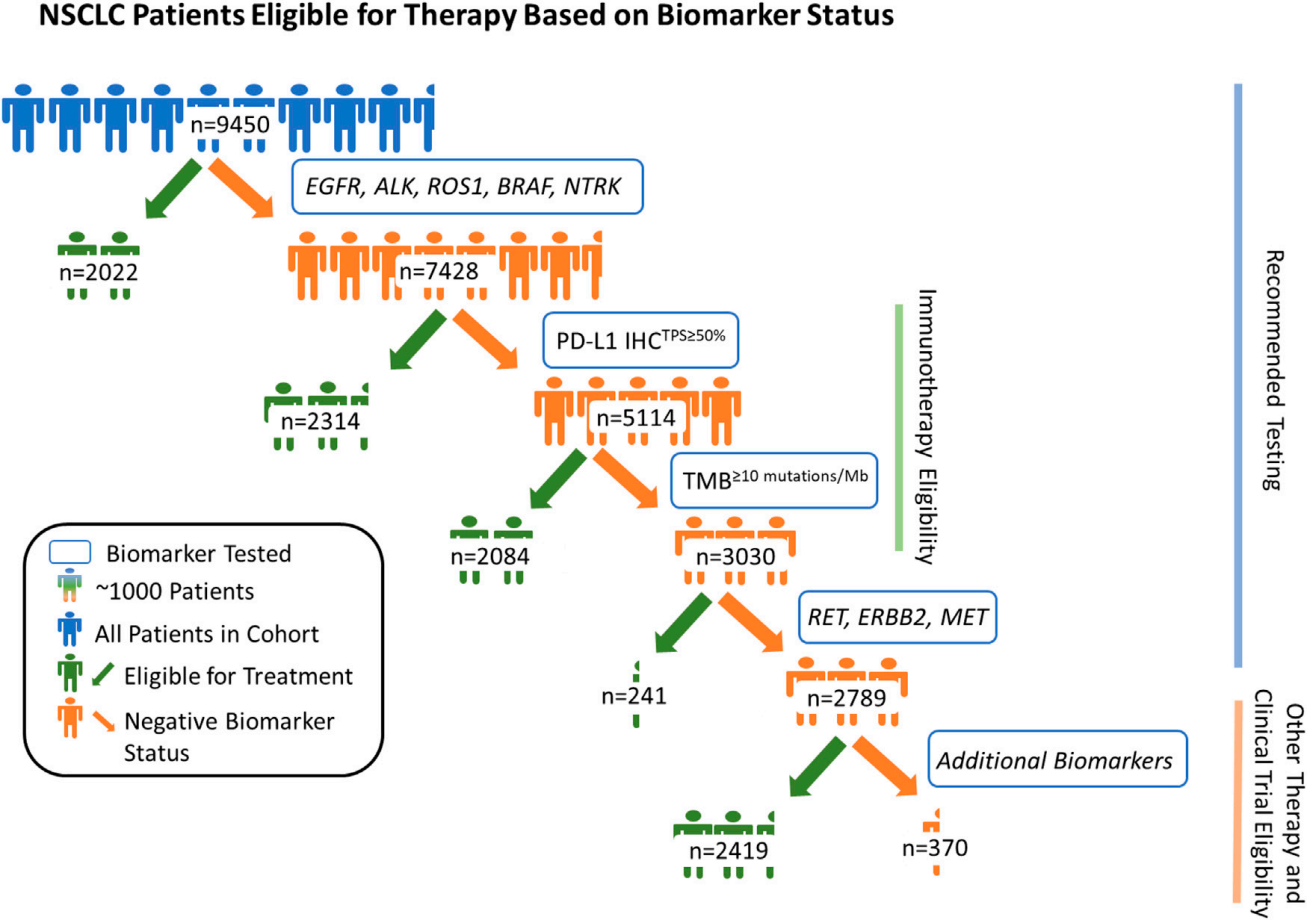
Patients with non-small cell lung cancer (NSCLC) eligible for therapy based on biomarker status. By assessing genomic driver alterations, tumor mutational burden (≥10 mutations/Mb cut-off), and PD-L1 expression (TPS ≥ 50% cut-off), we show that CGP + PD-L1 IHC yielded potentially actionable results, per National Comprehensive Cancer Network (NCCN) guidelines, for 70.5% of the 9,450 patients with NSCLC. Among the remaining 29.5% (2,789/9,450) of patients, 86.7% (2,419/2,789) were potentially eligible for another biomarker-associated therapy and/or clinical trial based on their genomic profile. In total, combined CGP and PD-L1 IHC testing provided positive biomarker statuses for 96.1% of 9,450 patients with NSCLC when considering potential eligibility for biomarker associated therapies and clinical trial enrollment.

Of the 9,450 samples tested, 2,022 patients (21.4%) were positive for a highly recommended NCCN recommended biomarker (*EGFR* (1,322/9,450) and/or *BRAF* (400/9,450) mutations; and/or *ALK* (228/9,450), *ROS1* (63/9,450) and/or *NTRK* (15/9,450) rearrangements). Of the remaining 7,428 patients, 2,314 (31.2%) were positive for PD-L1^TPS≥50%^ (Figure 1). Furthermore, 40.8% (2084/5,114) of the remaining patients without positivity in one of the previously mentioned biomarkers were TMB^≥10 mutations/Mb^. Next, of the patients that were not positive for one of the biomarkers mentioned above, 241 (8.0%) were positive for *RET* rearrangement (34/241), *ERBB2* mutations (98/241), *MET* mutations (79/241), and/or *MET* amplifications (37/241). In total, CGP + PD-L1 testing provided at least one positive NCCN recommended biomarker result for 70.5% (6,661/9,450) of NSCLC patients. Among the remaining patients without a positive NCCN recommended biomarker (*n* = 2,789), 86.7% (2,419) had an alteration which could be considered actionable or allow them to be potentially eligible for a biomarker-associated clinical trial (actionability associated with short variants, copy number alterations, and/or rearrangements in 90 genes deemed clinically relevant). Lastly, we examined the prevalence of *KRAS* mutations in the overall cohort and found that 28.9% (2,728/9,450) were positive for clinically relevant *KRAS* mutations and 11.8% (1,117/9,450) had *KRAS* G12C mutation.

### PD-L1 Tumor Cell Expression in NSCLC

**FIGURE 2:**
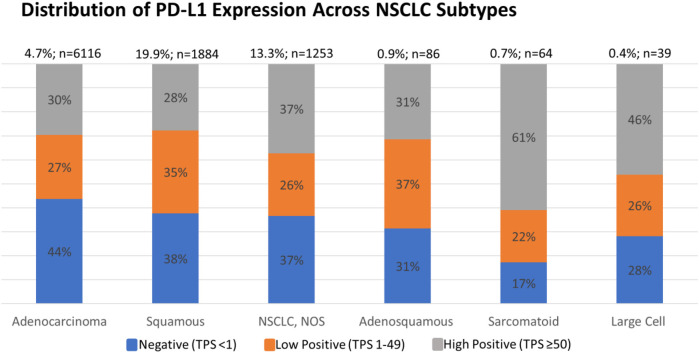
Percent of negative, low positive, and high positive PD-L1 cases of NSCLC subtypes. An increased high positive rate was detected in sarcomatoid carcinoma subtype vs. adenocarcinoma subtype (*p* = 0.025, *t*-test) and in large cell carcinoma subtype vs. adenocarcinoma subtype (*p* = 0.025, *t*-test).

Each PD-L1 IHC assay has its own immunotherapeutic agent-associated FDA approval. For NSCLC, the DAKO 22C3 assay is used for pembrolizumab as a companion diagnostic. Currently, pembrolizumab requires a TPS score of ≥1% for patients with NSCLC to be eligible for therapy (see Supplementary Figures S1, S2 for analysis based on TPS score of ≥1%) [2]. However, we selected a PD-L1^TPS≥50%^ in this analysis, as this was the previous FDA approved cut-off in NSCLC and represents the most conservative cut-off. We found that 30.5% of the 9450 NSCLC were PD-L1 TPS ≥ 50% and 28.3% were TPS 1–49%. In addition, we found a higher PD-L1^TPS≥50%^ positive rate in sarcomatoid carcinomas vs. adenocarcinomas (*p* = 0.025, *t*-test), and in large cell carcinomas vs. the adenocarcinomas (*p* = 0.025, *t*-test), subtypes of NSCLC (Figure 2).

### Immunotherapy Biomarkers Analysis

**FIGURE 3:**
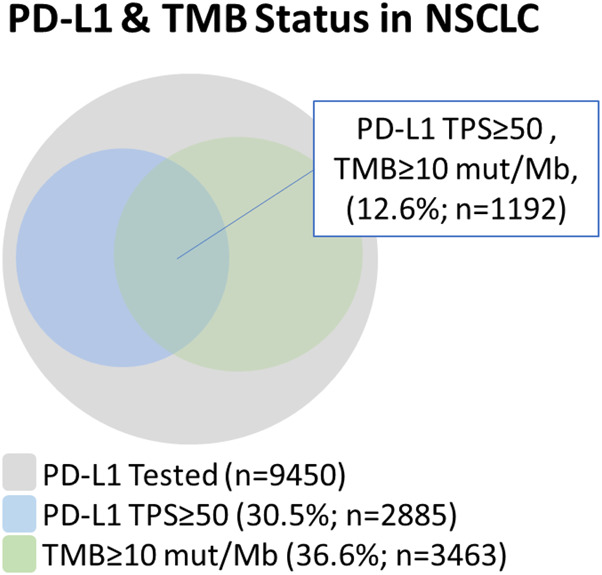
Relationship between PD-L1 and tumor mutational burden in NSCLC using a PD-L1 TPS ≥ 50% cut-off and a TMB ≥ 10 mutations/Mb cut-off.

Next, we examined the relationship between PD-L1^TPS≥50%^ with TMB^≥10 mutations/Mb^ status determined by CGP in NSCLC and its different subtypes (Figure 3). In NSCLC, of the 2885 PD-L1^TPS≥50%^, 41.3% (1,192/2,885) were also TMB^>10mutations/Mb^. This means that out of the 3463 TMB^>10mutations/Mb^, 65.6% (2,271/3,463) were not PD-L1^TPS≥50%^. Additionally, in our total patient cohort, 3.8% (36/9,450) of samples were MSI-H and of these patients, 0% (0/36) were PD-L1^TPS<50^/TMB^<10mutations/Mb^. The prevalence of potential immunotherapy resistance biomarkers in the PD-L1^TPS≥50%^/TMB^>10mutations/Mb^ cohort were 6.7% (80/1,192) *STK11* mutations, 6.2% (74/1,192) *KEAP1* mutations, and 3.1% (37/1,192) *EGFR* mutations.

### Genomic Alterations in the PD-L1 TPS ≥ 50% and TPS < 50% Disease Subsets

In the PD-L1^TPS≥50%^ disease subset, *BRAF* mutations, *MET* mutations, *MET* amplifications, and *KRAS* mutations were significantly enriched (*p* < 0.05, Fisher Exact Test with adjusted *p*-value). This contrasted with enrichment of *EGFR* mutations, *ERBB2* mutations, *STK11* mutations, and *KEAP1* mutations in the PD-L1^TPS<50%^ (*p* < 0.05, Fisher Exact Test with adjusted *p*-value) (Table 2).

**TABLE 2:** Prevalence of genomic alterations in the NSCLC PD-L1^TPS≥50^ and NSCLC PD-L1^TPS<50^ cohorts and comparison of the two groups using Fisher Exact Test with Bonferroni adjusted *p*-value.

	PD-L1 ≥ 50 (*n* = 2,885)	*n*	PD-L1 < 50 (*n* = 6,565)	*n*	Adjusted *p*-value
*EGFR* mutations	10.1%	292	15.7%	1,030	<0.001
*BRAF* mutations	5.6%	163	3.6%	237	<0.001
*ALK* rearrangements	3.0%	86	2.2%	142	0.236
*ROS1* rearrangements	0.9%	26	0.6%	37	0.886
*NTRK* fusions	0.1%	4	0.2%	11	1
*RET* rearrangements	0.8%	22	0.6%	41	1
*ERBB2* mutations	1.1%	32	2.0%	130	0.030
*MET* mutations	4.6%	133	1.4%	92	<0.001
*MET* amplifications	5.3%	153	1.6%	103	<0.001
*KRAS* mutations	36.7%	1,058	25.4%	1,670	<0.001
*STK11* mutations	6.2%	179	15.0%	982	<0.001
*KEAP1* mutations	4.9%	142	6.4%	417	<0.001

## Discussion

The use of CGP (TMB^>10mutations/Mb^) and PD-L1 IHC^TPS≥50%^ in NSCLC identified at least one positive NCCN recommended biomarker for 70.5% (6,661/9,450) of patients in this cohort. Among the remaining patients without a positive NCCN recommended biomarker (*n* = 2,789), 86.7% (2,419) had an alteration which could be considered actionable or allow them to be potentially eligible for a biomarker-associated clinical trial. In sum, this means that 96.1% (9,080/9,450) of patients tested had a positive result in at least one actionable or potentially actionable biomarker. For immunotherapy eligibility, by examining TMB^>10mutations/Mb^ and PD-L1^TPS≥50%^, an additional 2,271 patients were potential immunotherapy candidates when compared to PD-L1^TPS≥50%^ alone. These findings underscore the utility of combining CGP with IHC to expand potential eligibility for biomarker-associated therapies and clinical trial enrollment to most patients with NSCLC. Further investigation is necessary to determine if certain combinations of PD-L1 tumor cell expression and TMB status (i.e. PD-L1^TPS≥50%^ and TMB^>10mutations/Mb^) are associated with better response to immunotherapy when compared to a single biomarker test and its correlation with potential resistant immunotherapy biomarkers such as *STK11*, *KEAP1*, and *EGFR*.

In the PD-L1^TPS≥50%^ disease subset, *BRAF* mutations, *MET* mutations, *MET* amplifications, and *KRAS* mutations were significantly enriched. As there are available targeted therapies for *BRAF* mutations and *MET* genomic alterations, more clinical studies need to be conducted to assess the efficacy of combining immunotherapy with *BRAF* and/or *MET* inhibitors. Consistent with the literature, we saw evidence of high correlation between *KRAS* and PD-L1 expression [18]. As mentioned above, *KRAS* mutations have been associated with lack of response for *EGFR* TKIs and due to the paucity of *KRAS* targeted therapies in the past, *KRAS* mutant tumor have been hard to treat with targeted therapies. However, *KRAS* G12C is now a biomarker with promising targeted therapies (ClinicalTrials.gov Identifier: NCT03600883, NCT04006301, NCT03785249) and so immunotherapy combined with *KRAS* inhibitors should be explored further. In the PD-L1^TPS<50%^ cohort, *EGFR* mutations, *ERBB2* mutations, *STK11* mutations, and *KEAP1* mutations were enriched. *EGFR*, *STK11*, and *KEAP1* mutations are known potential resistance mutations for immunotherapy and the increased prevalence of these mutations in the PD-L1^TPS<50%^ cohort could suggest a reduced response to immunotherapy. In addition, while Guisier et al. demonstrated no decrease in immunotherapy response in *ERBB2* mutated NSCLC in a small cohort of patients, larger clinical studies need to be performed to further evaluate the role of *ERBB2* mutations in immunotherapy response [19].

One limitation of this study is that as a reference laboratory, clinical information including treatment history is not available for our patient cohort. However, in general, the samples we receive represent real-world samples often acquired from later stage diseases and that should be taken into consideration when interpreting the data. In addition, since we are a US based laboratory, most of samples are from patients of European ancestry. The rates of the NCCN guideline highly recommended biomarkers in our cohort were similar to the literature. For example, in our cohort, the rate of *EGFR* mutations was 14.0% (1,322/9,450), while in the literature the rate of *EGFR* mutations is approximately 15% for patients of European ancestry [20]. Of note, our PD-L1 expression rates were similar to certain pembrolizumab clinical trials rates of PD-L1 expression. For example, our TPS ≥ 50% prevalence was 30.5% compared to the 30.2% of the KEYNOTE-024 study [13].

In conclusion, these findings highlight the improved clinical utility of combining CGP with IHC to expand the biomarker-guided therapeutic options available for patients with NSCLC, relative to single biomarker testing alone.

## Data Availability

All datasets presented in this study are included in the article/Supplementary Material.
