# Supplementary material for: Landscape of Biomarkers in Non-small Cell Lung Cancer Using Comprehensive Genomic Profiling and PD-L1 Immunohistochemistry
Source: Pathol Oncol Res. 2021 Mar 11;27:592997. doi: 10.3389/pore.2021.592997 (PMC8262230; doi:10.3389/pore.2021.592997)

Supplement

Supplement Figure Legend

Supplement Figure 1. Patients with Non-small cell lung cancer (NSCLC) eligible for therapy based on biomarker status. By assessing genomic driver alterations, tumor mutational burden (≥ 10 mutations/Mb cut-off), and PD-L1 expression (TPS ≥ 1% cut-off), we show that CGP + PD-L1 IHC yielded potentially actionable results, per National Comprehensive Cancer Network (NCCN) guidelines, for 81.9% of the 9450 patients with NSCLC. Among the remaining 18.1% (1712/9450) of patients, 87.3% (1495/1712) were potentially eligible for another biomarker-associated therapy and/or clinical trial based on their genomic profile. In total, combined CGP and PD-L1 IHC testing provided positive biomarker statuses for 97.7% of 9450 patients with NSCLC when considering potential eligibility for biomarker associated therapies and clinical trial enrollment.

Supplement Figure 2. Relationship between PD-L1 and tumor mutational burden in NSCLC using a PD-L1 TPS ≥ 1% cut-off and a TMB ≥ 10 mutations/Mb cut-off.

Supplement Figure 1

NSCLC Patients Eligible for Therapy Based on Biomarker Status (analysis based on TPS ≥1% cut-off)

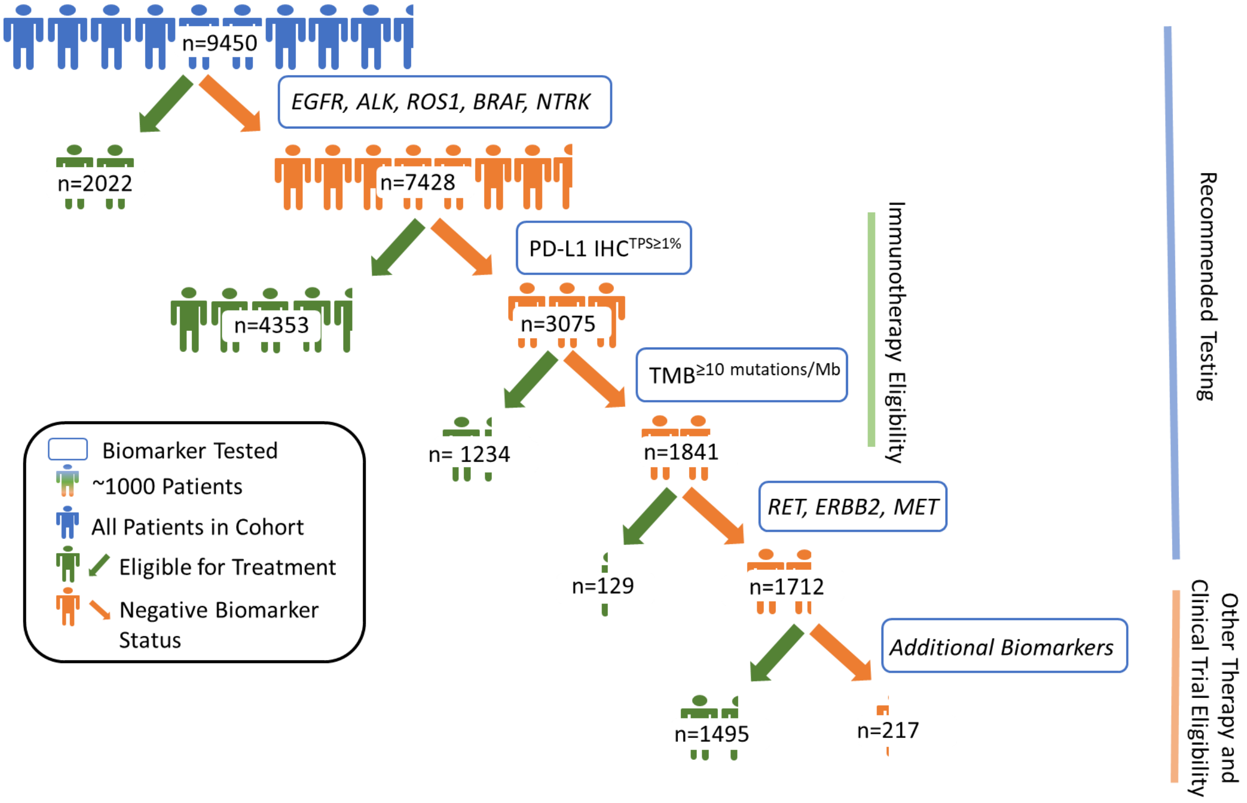

Supplement Figure 2

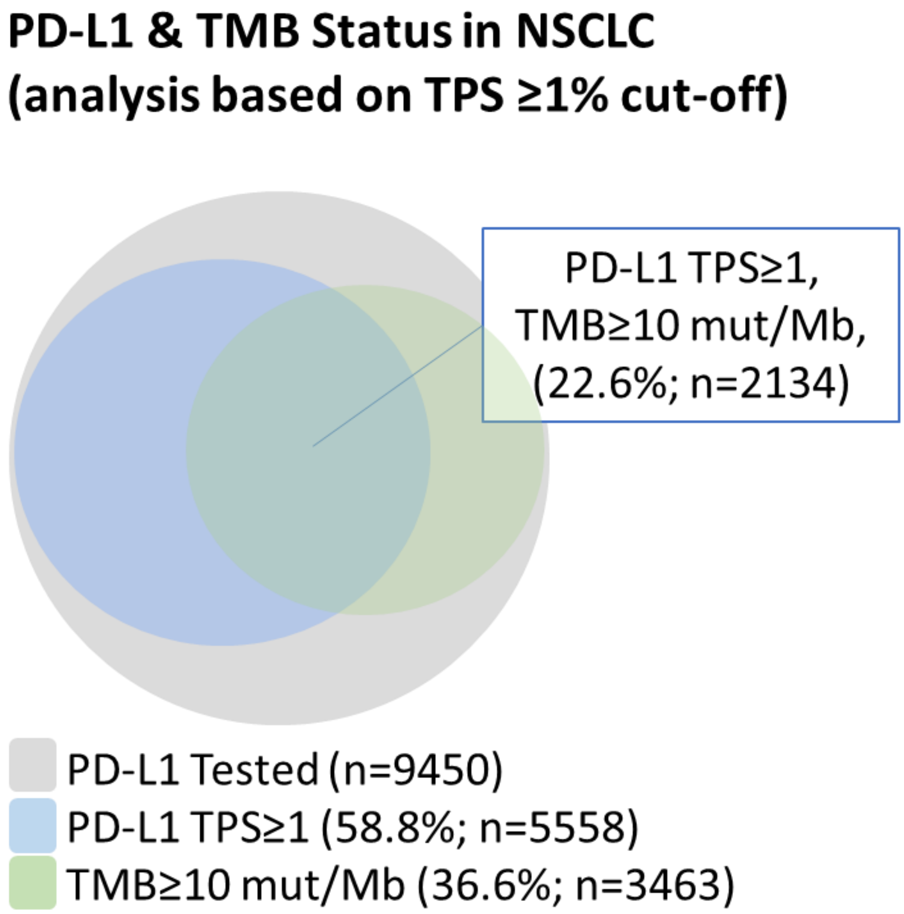

Supplement: Supplementary file 1 [file Image1.pdf]
